# Impact of salinity stress on cotton and opportunities for improvement through conventional and biotechnological approaches

**DOI:** 10.1186/s12870-023-04558-4

**Published:** 2024-01-02

**Authors:** Muhammad Tanees Chaudhary, Sajid Majeed, Iqrar Ahmad Rana, Zulfiqar Ali, Yinhua Jia, Xiongming Du, Lori Hinze, Muhammad Tehseen Azhar

**Affiliations:** 1 Horticultural Research Institute, National Agricultural Research Centre, Islamabad-44090, Pakistan; 2 Federal Seed Certification and Registration Department, Ministry of National Food Security and Research, Islamabad, 44090 Pakistan; 3 Center of Agricultural Biochemistry and Biotechnology/Centre of Advanced Studies in Agriculture and Food Security, University of Agriculture, Faisalabad, 38040 Pakistan; 4 Department of Plant Breeding and Genetics, University of Agriculture, Faisalabad, 38040 Pakistan; 5 State Key Laboratory of Cotton Biology, Institute of Cotton Research Chinese Academy of Agricultural Science, Anyang, 455000 China; 6 US Department of Agriculture, Southern Plains Agricultural Research Center, College Station, TX 77845 USA; 7 School of Agriculture Sciences, Zhengzhou University, Zhengzhou, 450000 China

**Keywords:** Biotechnology, Cotton, Development, Morphological, Physiological, Salinity

## Abstract

Excess salinity can affect the growth and development of all plants. Salinization jeopardizes agroecosystems, induces oxidative reactions in most cultivated plants and reduces biomass which affects crop yield. Some plants are affected more than others, depending upon their ability to endure the effects of salt stress. Cotton is moderately tolerant to salt stress among cultivated crops. The fundamental tenet of plant breeding is genetic heterogeneity in available germplasm for acquired characteristics. Variation for salinity tolerance enhancing parameters (morphological, physiological and biochemical) is a pre-requisite for the development of salt tolerant cotton germplasm followed by indirect selection or hybridization programs. There has been a limited success in the development of salt tolerant genotypes because this trait depends on several factors, and these factors as well as their interactions are not completely understood. However, advances in biochemical and molecular techniques have made it possible to explore the complexity of salt tolerance through transcriptomic profiling. The focus of this article is to discuss the issue of salt stress in crop plants, how it alters the physiology and morphology of the cotton crop, and breeding strategies for the development of salinity tolerance in cotton germplasm.

## Introduction

Salinity has exerted its impact on the productivity of field crops around the globe. It is regarded as serious threat for nations with economies based on agriculture. According to an estimate by the Food and Agriculture Organization of the United Nations [1], 8.7% of the world’s land (833mha) is effected by salinity and sodicity *i.e*., presence of a high proportion of sodium salt relative to other salts. The major causes of salinity in Pakistan are poor quality underground water, water logging, fertilizer intensive agriculture and sea water intrusion. The major cause in the disintegration and breakdown of arable land is poor irrigation practices. Published reports have indicated that salinity and sodicity reduce the cultivated land area available for agricultural production [2]. In Pakistan, salinity affects ~18% of the land in the Sindh province, 2% of the land in the Khyber Pakhtun Khawa province, and ~3% of the land in the Punjab province. Both primary and secondary cotton growing areas are present in these provinces. When cotton is grown in salinity affected soils in these provinces, cotton yields are very low. Therefore, it is crucial to take this issue seriously and develop sensible solutions to address salinity and sodicity in soils. This article highlights the effect of salt stress on crop plants in general, how it specifically alters the physiology and morphology of the cotton plant, and the strategies needed to develop salinity tolerance in cotton germplasm.

### Salinity: Hampered plant growth and development

The development and growth of the plants can be hampered by excessive salinity. Depending on a plant’s capacity to withstand excessive salt, some plants are impacted more than others [3]. Halophytes are plants that can withstand high soil salinity levels without experiencing a decrease in yield. Glycophytes, on the other hand are salt sensitive plants that exhibit poor performance under excess salt concentrations. Different plants have different levels of tolerance for salt stress. For instance, barley has been reported to tolerate salts up to an 8.5 dSm-1 threshold level, and soybeans have been reported to tolerate salts up to a 6.2 dSm-1 threshold level. Onions, however, have been found to be more sensitive to salt, only tolerating it up to 1.4 dSm-1 threshold level, equal to carrots' 1.6 dSm-1 threshold level [4, 5]. Cotton is considered as moderately salt tolerant crop among the cultivated crops in Pakistan [6]. Yield is unaffected by salt concentrations up to 7.7 dSm^−1^, but when salt concentrations reach 15 dSm^−1^, yield is reduced by 50% [7]. The quantity and quality of irrigation water, the kind of soil, the amount of irrigation water that evaporates, the temperature and density of the irrigation water, the surface geology, even the influx of salt water from the seas all affect salinity concentration. In Pakistan, majority of the soil is alkaline with little organic matter, and the subterranean water is unfit for irrigation, which leads to an increase in soil salinity. As the plants roots absorb water from the soil, these high salt concentrations cause physiological disruptions in the plant, including water uptake, water balance, accumulation of some solutes, K + uptake, disruption of ionic homeostasis, photosynthesis, respiration, gaseous exchange, transpiration, anatomy and morphology of the plant, and growth pattern [8]. The seedling, vegetative, and reproductive growth phases are effected by excessive salinity. Compared to a mature plant, seedlings are more vulnerable to salinity [9]. Salinity has put a drastic effect on cotton seed germination. The poor germination of seeds in the presence of excess salt can be due to two major phenomena: first, lower osmotic pressure leads to poor water imbibition capacity of seed water, and second, abnormal embryo development due to absorption of injurious ions from the soil. Very high concentrations of salt will inhibit germination [7, 10]. Plant growth is inhibited by salt stress for three reasons: 1) osmotic stress, leads to decrease in water potential in root zone and plants are unable to absorb the water they require [11], 2) specific ionic toxicity, where excessive both chloride and sodium ions are present and absorbed by the plant [12] and 3) nutrient deficiency, where plants are unable to absorb crucial nutrients like potassium, calcium, and nitrates [13]. When compared to crucial elements like K^+^ and Ca^++^ which are found in salt-affected soil at higher concentrations, Na + , Mg^++^, Cl^−^, and SO^4−^ are scarce and have a poor translocation rate inside the plant [14]. Sometimes too much sodium has an antagonistic effect and interferes the absorption of potassium and calcium. Under conditions of salt stress, calcium and potassium ions are crucial in the exclusion of Na^+^ from plant root cells through the plasma membrane [15]. Alfalfa has been shown to have higher salt tolerance in plants with a 20:1 ratio of Na^+^ to Ca^++^ [16]. Saline soils with high levels of sodium and chloride ions lead plants to absorb more of these ions, which results in ionic toxicity, notably from Cl- ions. Plants' cellular metabolic pathways are also disrupted by sodium and chloride ions [17]. Excessive sodium and chloride ions block the absorption of potassium ions, which are necessary for stomata to open and close and activate the cell's metabolic pathways. The growth of the plants is adversely effected due to absence of K^+^ that leads to the disruption of opening and closing of stomata. The high internal sodium and chloride concentration has been linked with reduced plant growth and low yield in many crops like mungbean [18], rice [19], wheat [20] and cotton [21]. Plants show reduced expansion of leaves caused by low water uptake [22]. Exclusion of Na^+^ ions occurs through anatomical organs in the plant cells [23].

At the beginning of salt stress, it has been documented that hormonal changes and the induction of reactive oxygen species (ROS) occur [24]. When stomata closure occurs to avoid water losses by transpiration, photosynthetic activities and carbon dioxide uptake tend to decrease. Plants exhibit aberrant root cell development, which causes a reduction in growth and development under salt stress and ultimately results in cell death [25]. The impact of salt stress on plants has wide range depending upon their growth stage. For instance, salt has a major impact on wheat plant growth during the early vegetative stage, but less so throughout the flowering and grain-filling stages [26]. Similarly, upland cotton plants suffer the most damage when they are in the seedling stage, whereas plants in the boll formation stage suffer the least damage [27]. Excessive salt affects the vegetative stage in strawberries, delaying flowering and fruit development [28].

### Damage to cotton due to salinity

Cotton is considered a moderately salt tolerant crop, but its yield is affected by low and abnormal germination in saline conditions. According to researchers, salinity adversely affects the stages of plant development as well as poor and aberrant plant germination [29]. A greenhouse experiment using potted plants was conducted using sodium chloride and sodium carbonate, and results showed that higher salt stress at the seedling stage disturbed plant morpho-physiological and biochemical attributes [30]. There is evidence to suggest that plant germination and yield decrease as salt stress increases. Additionally, the type of soil had a significant impact [31]. Silt and clay-rich soils had lower germination rates than sandy soils, which had higher germination rates. Salinity in silt and clay soils was thought to have a greater negative impact on the germination of cotton seeds because they have a higher capacity to retain harmful ions than sandy soils [32]. Physiological characteristics were used to assess the salinity tolerance of four genotypes of cotton (*Gossypium hirsutum* L.). Further, the ability of a cotton species to selectively absorb K^+^ by the activation of salt overly sensitive (SOS1), serine/threonine protein kinases (AKT1) and high affinity K^+^ transporter (HAK5) gene families also contributed to lower effects of salt stress [15]. The salt tolerance ability of diploid cotton species *G. klotzschianum* L. was assessed with the application of 300 mM NaCl for 0, 3, 12 and 48 h on roots. According to physiological research, levels of the hormone abscisic acid (ABA) increased significantly at 48 h while those of H_2_O_2_ did so at 12 h. However, at 48 h, there was a significant drop in glutathione (GSH) concentration. However, 37,278 unigenes were found in this experiment using transcriptome data, and 8,312 and 6,732 genes showed differential expression, indicating that they are involved in salt tolerance. According to gene function annotation and expression analyses, maintenance of ion homeostasis by ROS, hormone biosynthesis, and SOS1 gene signal transduction pathways ensures that cotton develops salt tolerance [33].

In order to determine the genetic basis of yield component, fiber quality, stomatal conductance, and plant architecture, a genome wide association study (GWAS) was carried out in cotton [34]. Cotton (*G. hirsutum* L.) genotypes were also explored based on Na^+^/K^+^ transport in leaves and hypocotyls as well as the relative abundance of salinity tolerant genes (GhSOS1, GhNHX1and GhAKT1) by using physiological and qPCR assays [14]. It was revealed that the concentration of K^+^ was higher than Na^+^ in leaves of salt tolerant cotton genotypes compared to salt sensitive genotypes. Further, an abundance of transcripts of GhSOS1 in leaves (controlling the Na^+^ ion extrusion and its transport from root to shoot) and GhAKT1 in hypocotyls (helping in K^+^ ion absorption and transport) were found in salt tolerant cultivars of rice [35]. An increase was also seen in concentration of the GhNHX1 transcript which encodes vacuole localized NHX protein, but no correlation was found between expression level and salt tolerance [36].

In maize and Arabidopsis, the ABP9 gene induces resistance to abiotic stress. It produces a bZIP transcription factor that binds to abscisic response element (ABRE2) motif of the maizecatalase1 gene. The ABP9 gene was transformed into cotton (*G. hirsutum* L.), and 12 transgenic lines were produced [37]. Under salt stress in the greenhouse, these lines were observed to have strong root systems, increased germination, reduced stomatal aperture, and stomatal diameter.

The ABP9 gene was overexpressed, which improved oxidative stress tolerance and reduced oxidative damage. The ABP9 gene increased the transcription of stress-related genes including GhGBP2, GhNCED2, GhZFP1, GhERF1, and GhHB1 within the transcriptome [38]. Using a principal component analysis, three groups of soybean cultivars-salt tolerant, moderately salt tolerant, and salt sensitive were developed against salinity stress. In comparison to sensitive genotypes and moderately salt tolerant genotypes, the salt tolerant genotypes had higher potassium content in the leaves than sodium as well as enhanced leaf area and water usage efficiency [5]. These results suggested that these parameters may also be used effectively for the validation of salt tolerance in other species of crop plants. Wheat varieties were evaluated for salinity tolerance with applications of potassium and zinc to plant potting medium in the greenhouse. According to the findings, potassium and zinc greatly reduced the severity of salt stress, and salt tolerant cultivars had greater fresh and dry biomass than control cultivars. Antioxidant enzymes (SOD, CAT, and APX) lose activity when exposed to salt stress, although their activity was noticeably increased in pots treated with zinc and potassium [39]. Other studies have shown that potassium applications can assist plants in overcoming salt stress [9].

## Breeding strategies for salinity tolerance

### Assessment of upland cotton accessions under salt stress conditions

#### Mediums of plant growth

Various types of media have been used for the screening of plants under salt stress [6]. Solution culture, hydroponic solution, and sand culture with salt solution were frequently employed as media in different studies [40]. In addition, some researchers have used pot culture with application of salt solution; petri dishes were also used with moistened filter paper containing a brine solution (10% NaCl) [41, 42].

#### Concentration of NaCl

Cotton can yield normally under electrical conductivity up to 7.7 dSm^−1^. To measure the limits of growth under higher salt concentrations, applications of salt stress at 10 dSm^−1^, 15 dSm^−1^ and 20 dSm^−1^ have been evaluated [43]. Further screening at a higher intensity of salt stress (25 dSm^−1^) has been applied by sowing the cotton crop in saline soil having electrical conductivity of 36.1 dSm^−1^ [44, 45]. However, some researchers have employed magnesium sulfate, sodium sulfate, and sodium chloride in various combinations to develop salt stress. NaCl has been widely utilized in screening for salt stress [46].

#### Estimation of genetic variability

The basic concept of plant breeding is to collect and explore the genetic variability for traits that a researcher wants to improve in crop plants [47]. Variation from high to low for parameters that enhance salt stress tolerance is pre-requisite for the selection of parent genotypes that are used in indirect selection or hybridization programs [48, 49]. Scientists have explored genetic diversity in tomato, rice, wheat, sorghum and maize which showed the presence of variation for salt tolerance related traits [50]. The stage of the crop when exposed to salt stress, the intensity of the salt stress and the type of germplasm evaluated by conducting screening experiment all affect how much variation is present for salinity tolerance [51]. In one study, the analysis of variance showed that data was non-significant under a lower level of salt stress, but it was significant when a higher level of salt stress was applied [52]. This literature review has identified that intra-specific and inter-specific variation was found among various lines of cotton under salt stress [53]. After the confirmation of variations within the crop, three approaches may be used to develop improved germplasm [54]. First, examine the presence of salt tolerance in germplasm and directly grow the lines that are tolerant to salinity [55]. The second approach involves the screening of genetic material (developed from cross pollination or self-pollination or already available accessions) under salt stress, and the genotypes that revealed good performance should be selected for future breeding methods [56]. The final strategy involves the identification of wild relatives that can tolerate salt, so that genes from these relatives can be transferred into present genetic stock for the development of salt tolerance through hybridization and transgenic crop breeding [57].

#### Gene action in saline environments

Salt tolerance mechanism is a complex process and involving multiple genes and interactions between genetic, developmental, and physiological factors [58]. However, there is still some ambiguity because while it has been suggested that crop plants' salt tolerance may be caused by minor genes, other research has found that salt tolerance is controlled by a single dominant gene [59]. In a maize salt tolerance study, difference of presence/absence of a single gene (glycine betaine) between the tolerant and susceptible lines were present [60]. The range of variation in germination percentage data in another study revealed the existence of polygenic inheritance [61]. Exclusion of chloride (Cl^−^) from progeny of crosses between cultivars of soybean indicated that a single dominant gene controls the inheritance of Cl^−^ exclusion [62]. In contrast, literature also revealed that exclusion of Cl^−^ from shoots of some woody perennial species was due to polygenic inheritance [63]. Research also revealed the existence of non-additive and additive types of genetic behavior for salinity tolerance in soybean [64], wheat [65], tomato [66] and pearl millet [67]. Salt tolerance was governed by non-additive gene action in maize [68]. In contrast, the salt tolerance phenomenon in sorghum was governed by additive genes, partial dominance and dominance [69–71].

### Basis of selection for salt tolerance

#### Statistical models

The capacity of crop plants to withstand against stress was analyzed by using various statistical models like absolute and relative models that are used as selection criterion for identifying salt tolerant genotypes [72, 73]. A salinity tolerance index has also been employed to differentiate between salt tolerant and sensitive genotypes [74].

### Useful traits for plant selection

#### Morphological traits

Plant morphologic parameters such as plant height, root length, shoot length, root weight, shoot weight and seedling vigour traits, including root and shoot length [10], fresh root and shoot weight, dry root and shoot weight, seedling vigor and dry mass are used as selection criteria for salt tolerance [75, 76].

### Physiological and biochemical traits

Numerous physiological markers have been approved for use in genetic screening for salt tolerance. Commonly used parameters in field conditions include stomatal conductance [77], chlorophyll content [78], proline and osmolytes, leakage of electrolytes from leaf discs [20], Na^+^ and Cl^−^ exclusion, and potassium to sodium ratio [17]. Recently, relative leaf water content (RLWC) also was proven to be a good selection criterion for the salt tolerance of cotton [79]. Plants primarily combat oxidative stress using an internal defense system made up of many enzymatic (SOD, CAT, POD, APX, GR, MDHAR, DHAR, GPX, GOPX, GST, AOX, PRXs, TRXs, GRX, etc.) and nonenzymatic antioxidants (AsA, GSH, ascorbic acid, phenolic acids, flavonoids, carotenoids, α-tocopherol, etc.). Under salt stress conditions, H_2_O_2_ is produced in cells which leads to the formation of ROS. The ROS damages the various organelles of plant cells [80]. The total soluble protein content in plant cells decreases due to the increase in salt concentration. H_2_O_2_ synthesis activates the cell defense system, which results in the production of antioxidants. The production of antioxidants has been used as an effective criterion to identify plants showing salt tolerance. CAT is essential in the detoxification of hydrogen peroxide into water and oxygen [81]. Increased H_2_O_2_ generation initiates a cascade of events that leads to the increased level of SOD within the plant cell. Salt tolerant genotypes showed a higher quantity of SOD under salt stress than sensitive genotypes. It decreased the toxic effect of ROS in plant leaves and root cells. Peroxidase (POD) also served the purpose of detoxifying H_2_O_2_ effects inside of the cytosol, chloroplast, and vacuoles. H_2_O_2_ was likewise transformed into water and molecule oxygen by it [82]. The presence of above mentioned antioxidants are important in regulating plant stress responsive mechanism for scavenging H_2_O_2_ which ultimately reduces the toxic effect of salt in plants. The prescenece of POD has been found higher in salt tolerant accessions whereas plants susceptible to salt stress have lower quantities of antioxidants [83]. APX is an important enzyme found abundantly in algae and plants [84]. The main function of APX is to scavenge H_2_O_2_ in the cytosol and chloroplasts, while CAT mainly functions in the peroxisomes. Because APX is more widely distributed and has a significant affinity for H_2_O_2_, it is more effective as an H_2_O_2_ scavenger under salt stress conditions than CAT. The oxidoreductase class of enzymes includes GR, which requires NADPH as a reducing agent. The cytosol, mitochondria, and chloroplasts contain the majority of the GR isoforms [85]. The GR enzyme protects cells against ROS by converting GSSG to GSH with the complementary oxidation of NADPH and restoration of GSH concentration. GPX is mostly localized in the chloroplast; however, various isoforms are located in the mitochondria, cytosol, and peroxisomes [86]. The GPX enzyme is considered a key enzyme in the removal of H_2_O_2_ as it functions both outside and inside the cell organelles. MDHAR has several isoforms that are present in organelles such as mitochondria, chloroplasts, glyoxysomes, peroxisomes, and the cytosol. Together with other antioxidant enzymes such as SOD, APX, MDAR, and GR, MDHAR ensures the completion of the Foyer-Asada-Halliwell pathway and the removal of ROS in chloroplasts and cytoplasm. The accumulation of non-enzymatic antioxidants like α-tocopherol [87], carotenoids [88], ascorbic acid [89] and polyphenols [90] has been shown to be higher in halophytes than in glycophytes. Taken together, regulation of enzymatic and nonenzymatic components of the antioxidant system enables halophytes to protect themselves against oxidative damage. Therefore, the role of antioxidant is very important for the selection of salt tolerant genotypes in cotton [91].

### Molecular basis for salt tolerance

**Fig. 1:**
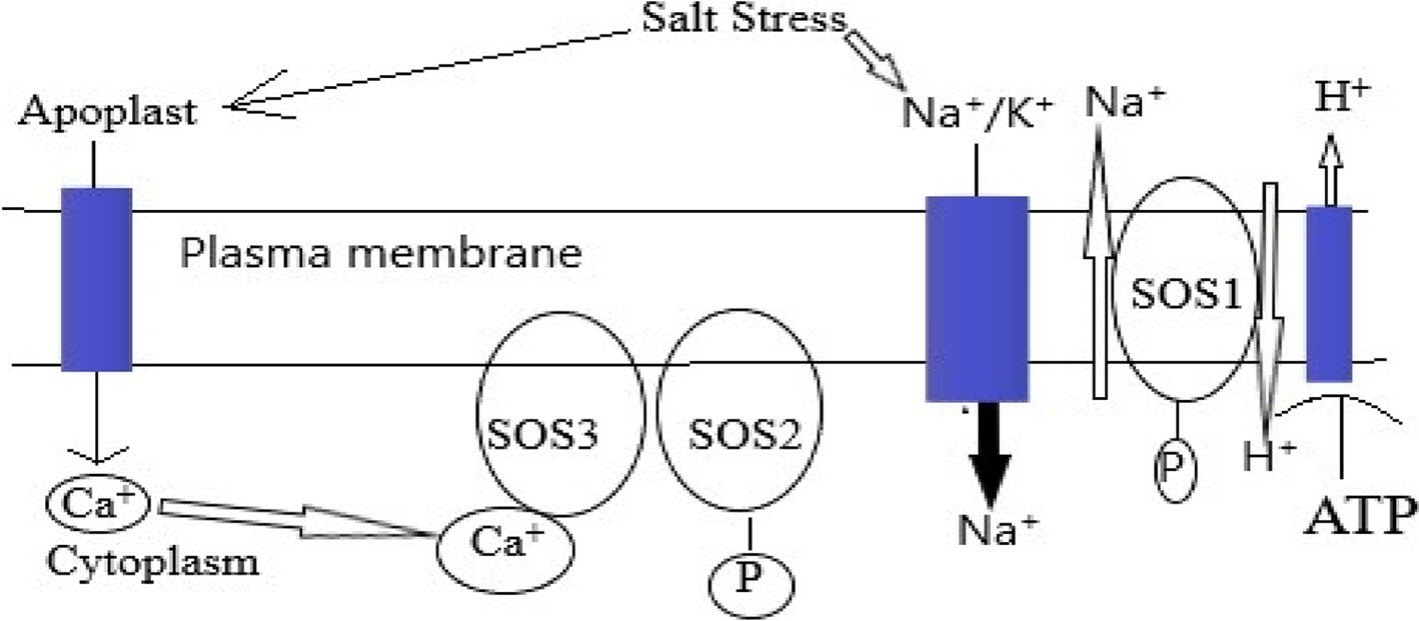
Salt overly sensitive (SOS) stress signaling pathways that lead to tolerance of sodium ions in cotton

It is difficult to understand the genetic basis of complex traits which are controlled by many genes. Because tolerance is based on several parameters and is not completely understood at the molecular level, there has been limited success in the development of salt tolerant genotypes in cotton. However, advancement in biochemical and molecular research approaches has made it possible to explore complex traits through transcriptomic profiling under salt stress. The inclusion of transcriptomic analysis facilitate the scientist to develop salt tolerant accessions in *Arabidopsis* [20] and tobacco [92]. At the molecular level, salt tolerance mechanisms fall into two categories: osmotic homeostasis and ionic homeostasis. When these two systems work together, secondary stresses emerge [93]. The transportation of sodium and potassium ions in plant cells are possible through ionic homeostasis. However, the role of chloride ions is still not clear in homeostasis. One of the mechanisms relies on salt overly sensitive (SOS) stress signaling pathways that leads to tolerance of sodium ions [94] (Fig. 1). The H^+^ pumps play a major role in ionic homeostasis, Na^+^ influx channels and sodium ions compartmentalization in vacuoles [95]. There are two types of H^+^ pumps: ATPase and ATPase in plasma membrane.

Specific transporters facilitates Na^+^ to cross cell membrane such as low affinity cation transporters (LCT) and high affinity potassium transporters (HKT) [21]. It has also been suggested that some non-selective cation channels (NSCC) play a role in the long-distance transfer of sodium ions to the shoots in plants. The Na^+^/H^+^ (NHX) antiporters, specifically SOS1, facilitate several sodium ion transport pathways that move sodium across the plasma membrane. This antiporter family is thought to aid in the compartmentalization of Na^+^ ions in vacuoles, which results in salinity tolerance [96]. The NHX family and high expression of SOS1 genes, combined with a cell-specific promoter and regulators, are used to developed genotypes that are salt tolerant [97]. The saline solution leads to ionic imbalance between apoplast and symplast that results in loss of turgor pressure [98]. When the turgor pressure was below the capacity of the cell wall, plant development ended. Plants accumulate numerous osmolytes and osmoprotectants to maintain osmotic homeostasis [99]. The expansion in vacuolar and cell volume is due to compartmentalization of injurious ions and the accumulation of osmoprotectants in plant cells [31]. The osmoprotectants are organic and consist of glycine betaine, fructose, trehalose, proline and glucose. These play an important role in maintaining plasma membrane and thylakoid integrity, preventing lipid peroxidation and maintaining the structure of various protein molecules through detoxification of ROS (*e.g*., H_2_O_2,_ proline and POD) [100, 101]. The role of calcium ions and H_2_O_2_ in the signaling of cytosol is important because they exert stress and induce the secretion of abscisic acid (ABA), leading to stomatal closure and activation of genes for salinity tolerance [102]. The over expression of genes under salt stress facilitated the plants to cope with salt stress conditions. It is also facilitated the development of salt tolerant accessions, [103]. Similarly, the gene for glycine betaine coda, β glycine betaine, produced more choline oxidase, choline dehydrogenase and betaine aldehyde dehydrogenase [104]. Besides the cited genes, HVA1, Di and DREB gene families are involved in drought and salinity tolerance in crop plants [105]. The role of Cys2/His2-type zinc-finger proteins GhDi 19–1 and GhDi 19–2 is crucial to avoid cell dehydration and integrity [37, 106, 107]. Some of the genes related to salinity tolerance in cotton are given in Table 1.

**Table 1:** Summary of genes related to salinity tolerance in cotton and their functions

Sr #	Genes	Function	Reference
1	*GhDREB*	Dehydration responsive element binding protein	[108]
2	*GhERF6*	ERF encoding genes	[109]
3	*GhNHX1*	Tonoplast Na/H antiporter	[110]
4	*GhMT3a*	Type 3 metallothione in protein	[111]
5	*GhMPK2*	Mitogen-activated protein kinase	[112]
6	*GhNAC1*	Encode NAC domain	[113]
	*GhNAC4*		
	*GhNAC6*		
7	*GhSOD1*	Superoxide dismutase	[114]
8	*GhDi19-1*	Drought induced protein which is Cys2/His2 zinc-finger protein	[115]
	*GhDi19-1*		
9	*GhWRKY11*	WRKY transcription factor	[116]
	*GhWRKY12*		
	*GhWRKY13*		
	*GhWRKY14*		
	*GhWRKY15*		
	*GhWRKY20*		
	*GhWRKY21*		
	*GhWRKY24*		
	*GhWRKY30*		
	*GhWRKY32*		
	*GhWRKY33*		
	*GhWRKY34*		
10	*GhAnn1*	Annexin gene	[117]
11	*GhCCL*	Cold circadian rhythm-RNA binding-like protein	[118]
12	*GhTPS11*	Trehalose-6-phosphate synthase	[119]
13	*GhABF2*	bZIP-encoding gene	[120]
14	*GhZAT34*	Genes of zinc finger proteins	[121]
	*GhZAT79*		

### Mechanisms of salinity tolerance

**Fig. 2:**
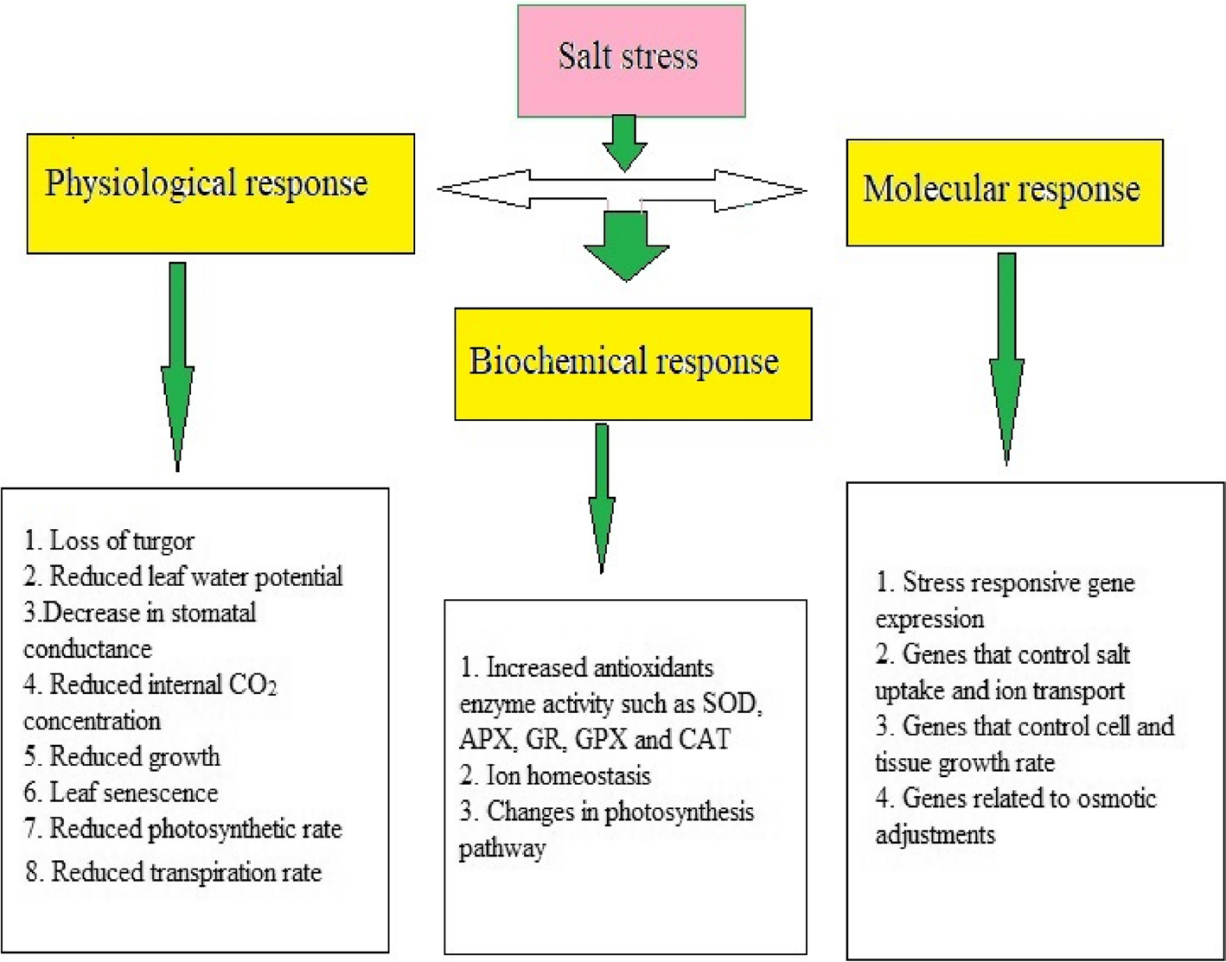
Physiological, biochemical and molecular responses of crop plants against salt stress

Some plants growing in salt stress environments have been observed to adapt to decreased water potential and high Na^+^ toxicity in order to sustain normal growth [122]. Furthermore, adjustment of ionic balance for maintaining homeostasis is an important parameter in plants [123, 124]. The exclusion of injurious ions and less movement of Na^+^ inside the cell prevent it from a high level of damage [6]. The development of salt tolerance is a phenomenon that also occurs in glycophytes. To develop high salt tolerance, plants use two different strategies: 1) morphological approach like succulence and the presence of structures that control salt stress, and 2) physiological strategies like ion selection, ionic compartmentalization, and osmolyte production [22]. In addition, various metabolic processes that include osmotic potential, nutritional deficiencies and ionic toxicity are occurring inside the cotton plant that negatively affect their growth under salt stress. Among these stress inducing factors, the primary factor is nutritional imbalance, and the secondary factors are osmotic potential and ionic toxicity [125]. Plants undergo physiological drought when their osmotic potential drops due to high salt levels. Furthermore, salinity increases the level of Na^+^ in soil which produces injurious ions and additionally causes a lack of essential minerals, resulting in reduced plant development and production [15]. Figure 2 summarizes the physiological, biochemical and molecular responses of crop plant towards salt stress.

### Physiological mechanisms

#### Succulence

Plants that use a lot of water can balance ionic toxicity in salty environments. These plants are classified as succulents because they absorb a lot of water [126]. Succulence generally involves increased cell size, increased leaf water content per unit area and decreased growth and cell suspension [127]. In terms of physiology, the cotton plant's high water intake helps in diluting the harmful ions, resulting in a high level of salt tolerance. Plants can tolerate salt stress due to the thickness of leaves coupled with increased mesophyll cell size [12, 128]. As a result, there are more mitochondria, which means there is more energy available for salt compartmentalization and Na^+^ exclusion [129].

### Specialized structures

Some halophytes have evolved specific structures throughout time that reduce their susceptibility to salt stress [130]. These structures, including bladders, trichromes and salt glands, work as storehouses under saline conditions. Besides succulence, cotton plants have evolved some structural changes like fewer and smaller leaves to cope with salt stress. The salinity tolerant mechanism involved the root lignification and the existence of wax on epidermis in plants [2, 131].

### Ionic compartmentalization

Vacuoles are largely used in ionic compartmentalization in leaves to store salts reducing the number of harmful ions [132]. Salt-tolerant plants may store three times as much salt in their bodies as normal plants. The maintenance of less injurious ions by the salt tolerant cultivars in the cytosol controlled the metabolic activities in chloroplast and phloem, thus the process of photosynthesis is not disturb [117, 133]. Furthermore, it has been observed that due to the compartmentalization of salts in vacuoles, significant salt accumulation in cells does not disrupt the photosynthetic activities [134]. Additionally, Na^+^ and Cl^−^ ions can be delivered and accumulate in particular tissues through a variety of distinct channels without interfering with metabolic activities [24].

### Selective ionic transportation

Cotton plants that are salt tolerant have distinct routes that allow them to absorb certain ions and deliver them to different parts of the plant. When the K^+^/Na^+^ ratio deviates from normal, many metabolic processes and physiological pathways are disrupted [24]. Different types of transporters and proton pumps are used to transfer K^+^/Na^+^ into plants. As a result, a plant's ability to maintain K^+^/Na^+^ inside the cell can be considered as a selection criterion for tolerance to saltwater [25]. Plant antiporters have been identified which are responsible for reducing Na^+^ toxicity within plant cells as well as potassium ion absorption [27]. Some plants have various ion transport systems that assure the existence of a high potassium ratio, which confers salt tolerance [135].

### Compatible solutes

Certain substances have a negative impact on the soil's water permeability in the root zone, which limits the amount of water that can reach the plants [136]. To cope with these damages, plants have developed a phenomenon where osmolytes are secreted to adjust osmotic balance [18]. Using this method, cellular sub-organelles remain protected and oxidative damage inside the plants is reduced [137, 138]. Polyols, sugars, mannitol, ammonium and sulfur compounds, and amino acids are examples of osmolytes. Polyamine, glycine, cholines and proline have all been found to aid in plants' ability to tolerate salt stress. More than 80 times increased proline level has been found in tobacco exposed to high salt concentrations as compared to the control [139]. When plants are subjected to salt stress, a large production of abscisic acid has been observed to maintain K^+^/Na^+^ equilibrium in cotton [140].

### Recent advances in cotton for salinity tolerance

**Fig. 3:**
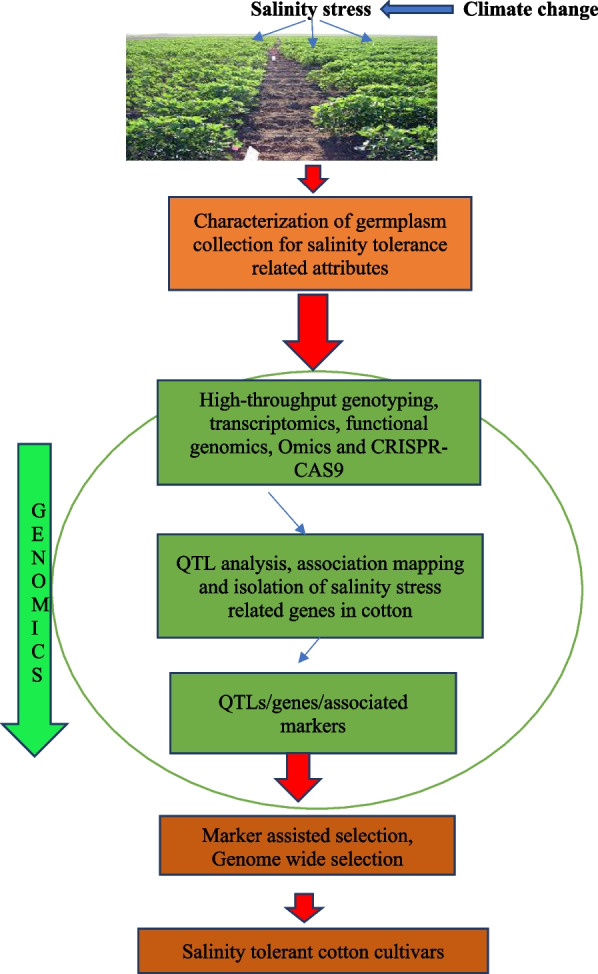
Schematic diagram for the development of salinity tolerant cotton cultivars using modern molecular approaches

The presence of phenotypic variation in response to increasing salt concentrations and subsequently exploring its genetic basis may lead to the development of salt tolerant germplasm in cotton [141]. In this modern era of molecular genetics, various transcription factors were found that are considered important regulators of gene expression. Several salinity tolerant genes have been reported in crop plants. However, in cotton, few genes related to salt tolerance have been identified. Those identified include *ERF* [142], tonoplast *Na*^+^*/H*^+^ *antiporter* [143], *GhMT3a* [144], *ZFP* [145], *NAC* [146], *DREB* [147], *MPK* [86] and *MKK*. Fan et al*.* [148] identified 108 *WRKY* genes (*GarWRKYs*) in *G. aridum* using transcriptome sequencing data, and real time-polymerase chain reaction (RT-PCR) analysis confirmed the expression of 26 *GarWRKY* genes in the roots. Overexpression of ROS scavengers like *GhCAT1, GhSOD1* and *GhMT3a* was observed in various cotton lines showing tolerance to salt stress. The expression of the novel gene (*GhNHX1)* regulates the Na^+^/H^+^ antiporter, tonoplasts and defense responses against salt stress. Expression analysis showed that mRNA level of *GhNHX1* was higher in salt tolerant genotypes as compared to sensitive genotypes, suggesting its importance in the salt tolerance mechanism. Genome modification through targeted genome editing has revolutionized plant genomics for improving plants’ characteristics against biotic and abiotic stressors [149]. Mega nucleases, transcription activator-like effector nucleases (TALENs), zinc finger nucleases (ZFNs), and clustered regularly interspaced short palindromic repeats (CRISPR/Cas) systems have all been used to alter genomes. CRISPR–Cas9 has been widely utilized to transform plants by inducing mutations via either homology-dependent repair (HDR) or non-homologous end-joining of double-stranded breaks (NHEJ) [150]. The following Fig. 3 shows the importance of modern molecular approaches in the development of salt tolerance cultivars of cotton.

## Conclusion

In conclusion, salinity is a serious problem which poses a threat to ensuring food security as more than half of the countries in the world are facing this problem. Salt stress causes toxicity of specific ions and induces water stress and nutrient imbalance in plant tissues which disturbs plant growth and development. Salt stress affects the metabolic activities of enzymes, impairs nutrient uptake and results in nutritional disorders which leads to yield reduction and fiber quality deterioration in cotton. High-throughput and efficient modern molecular approaches are important for salt stress genetic screening. CRISPR-CAS9 and genomics approaches have been shown to be quick and effective ways for exploring the molecular regulation of plant salt tolerance. Transcriptome sequencing techniques have also been frequently employed to discover new genes involved in the control of plant salt stress responses. Development of salt tolerant cotton cultivars offers a management strategy to control the negative salt stress response in cotton. To develop these cultivars, it is crucial to form collaborations joining the skills and expertise of conventional plant breeding and modern molecular approaches.

## Data Availability

Not applicable.

## References

[1] Fao F (2021). Agricultural and rural extension worldwide: options for institutional reform in the developing countries.

[2] Yadav S, Irfan M, Ahmad A, Hayat S (2011). Causes of salinity and plant manifestations to salt stress: a review. J Environ Biol.

[3] Blum A (2018). Plant breeding for stress environments.

[4] Dhivya R, Amalabalu P, Pushpa R, Kavithamani D (2014). Variability, heritability and genetic advance in upland cotton (*Gossypium hirsutum* L.). Afric J Plant Sci.

[5] Shelke D, Pandey M, Nikalje G, Zaware B, Suprasanna P, Nikam T (2017). Salt responsive physiological, photosynthetic and biochemical attributes at early seedling stage for screening soybean genotypes. Plant Physiol Biochem.

[6] Sikder RK, Wang X, Jin D, Zhang H, Gui H, Dong Q, Pang N, Zhang X, Song M (2020). Screening and evaluation of reliable traits of upland cotton (*Gossypium hirsutum* L.) genotypes for salt tolerance at the seedling growth stage. J Cotton Res.

[7] Yue H, Mo W, Li C, Zheng Y, Li H (2007). The salt stress relief and growth promotion effect of Rs-5 on cotton. Plant Soil.

[8] Ismail AM, Horie T (2017). Genomics, physiology, and molecular breeding approaches for improving salt tolerance. Ann Rev Plant Bio.

[9] Jan AU, Hadi F, Nawaz MA, Rahman K (2017). Potassium and zinc increase tolerance to salt stress in wheat (*Triticum aestivum* L.). Plant Physiol Biochem.

[10] Riaz M, Farooq J, Sakhawat G, Mahmood A, Sadiq M, Yaseen M (2013). Genotypic variability for root/shoot parameters under water stress in some advanced lines of cotton (*Gossypium hirsutum* L.). Genet Mol Res.

[11] Sevik H, Cetin M (2015). Effects of water stress on seed germination for select landscape plants. Pol J Environ Stud.

[12] Shabala S (2000). Ionic and osmotic components of salt stress specifically modulate net ion fluxes from bean leaf mesophyll. Plant Cell Environ.

[13] Read JJ, Reddy KR, Jenkins JN (2006). Yield and fiber quality of upland cotton as influenced by nitrogen and potassium nutrition. Europ J Agron.

[14] Peng Z, He S, Sun J, Pan Z, Gong W, Lu Y, Du X (2016). Na^+^ compartmentalization related to salinity stress tolerance in upland cotton (*Gossypium hirsutum*) seedlings. Sci Rep.

[15] Wang N, Qiao W, Liu X, Shi J, Xu Q, Zhou H, Yan G, Huang Q (2017). Relative contribution of Na^+^/K^+^ homeostasis, photochemical efficiency and antioxidant defense system to differential salt tolerance in cotton (*Gossypium hirsutum* L.) cultivars. Plant Physiol Biochem.

[16] Wei TJ, Jiang CJ, Jin YY, Zhang GH, Wang MM, Liang ZW (2020). Ca2+/Na+ ratio as a critical marker for field evaluation of saline-alkaline tolerance in alfalfa (*Medicago sativa* L.). Agronomy.

[17] Liu X, Cai S, Wang G, Wang F, Dong F, Mak M, Holford P, Ji J, Salih A, Zhou M (2017). Halophytic NHXs confer salt tolerance by altering cytosolic and vacuolar K+ and Na+ in *Arabidopsis* root cell. Plant Growth Reg.

[18] Saha P, Chatterjee P, Biswas AK (2010). NaCl pretreatment alleviates salt stress by enhancement of antioxidant defense system and osmolyte accumulation in mungbean (*Vigna radiata* L. Wilczek). Ind J Exp Biol.

[19] Ahmad H, Zafar SA, Naeem MK, Shokat S, Inam S, Rehman MA, Naveed SA, Xu J, Li Z, Ali GA, Khan MR (2022). Impact of pre-anthesis drought stress on physiology, yield-related traits and drought responsive genes in green super rice. Front Gen.

[20] Shokat S, Großkinsky DK, Roitsch T, Liu F (2020). Activities of leaf and spike carbohydrate-metabolic and antioxidant enzymes are linked with yield performance in three spring wheat genotypes grown under well-watered and drought conditions. BMC Plant Biol.

[21] Taghizadeh N, Ranjbar GA, Nematzadeh GA, Ramazani Moghaddam MR (2018). Salt-related genes expression in salt-tolerant and salt-sensitive cultivars of cotton (*Gossypium* sp. L.) under NaCl Stress. J Plant Mol Breed.

[22] Silva ED, Ribeiro R, Ferreira-Silva S, Viégas R, Silveira J (2010). Comparative effects of salinity and water stress on photosynthesis, water relations and growth of Jatropha curcas plants. J Arid Environ.

[23] Urano K, Kurihara Y, Seki M, Shinozaki K (2010). ‘Omics’ analyses of regulatory networks in plant abiotic stress responses. Curr Opinion Plant Biol.

[24] Roy SJ, Tucker EJ, Tester M (2011). Genetic analysis of abiotic stress tolerance in crops. Curr Opinion Plant Biol.

[25] Toyoshima C, Kanai R, Cornelius F (2011). First crystal structures of Na^+^, K^+^-ATPase: new light on the oldest ion pump. Structure.

[26] Carillo P, Annunziata MG, Pontecorvo G, Fuggi A, Woodrow P (2011). Salinity stress and salt tolerance. *Abiotic Stress in Plants-Mechanisms and Adaptations*. INTECH.

[27] Blumwald E, Aharon GS, Apse MP (2000). Sodium transport in plant cells. Biochimica et Biophysica Acta (BBA)-Biomembranes.

[28] Tremblay AM, Dufour CR, Ghahremani M, Reudelhuber TL, GiguèRe V (2010). Physiological genomics identifies estrogen-related receptor α as a regulator of renal sodium and potassium homeostasis and the renin-angiotens in pathway. Mol Endocrin.

[29] Gumz ML, Rabinowitz L, Wingo CS (2015). An integrated view of potassium homeostasis. NewEngland J Med.

[30] Shabala S, Cuin TA (2008). Potassium transport and plant salt tolerance. Physiol Plant.

[31] Ahmad S, Khan N, Iqbal MZ, Hussain A, Hassan M (2002). Salt tolerance of cotton (*Gossypium hirsutum* L.). Asian J Plant Sci.

[32] Dong H, Li W, Tang W, Zhang D (2009). Early plastic mulching increases stand establishment and lint yield of cotton in saline fields. Field Crops Res.

[33] Wei D, Zhang W, Wang C, Meng Q, Li G, Chen TH, Yang X (2017). Genetic engineering of the biosynthesis of glycine betaine leads to alleviate salt-induced potassium efflux and enhances salt tolerance in tomato plants. Plant Sci.

[34] Gapare W, Conaty W, Zhu Q-H, Liu S, Stiller W, Llewellyn D, Wilson I (2017). enome-wide association study of yield components and fibre quality traits in a cotton germplasm diversity panel. Euphytica.

[35] Kumar V, Singh A, Mithra SA, Krishnamurthy S, Parida SK, Jain S, Tiwari KK, Kumar P, Rao AR, Sharma S (2015). Genome-wide association mapping of salinity tolerance in rice (*Oryza sativa* L.). DNA Res.

[36] Qin F, Shinozaki K, Yamaguchi-Shinozaki K (2011). Achievements and challenges in understanding plant abiotic stress responses and tolerance. Plant Cell Physiol.

[37] Hopkins GR, Brodie ED, Neuman-Lee LA, Mohammadi S, Brusch Iv GA, Hopkins ZM, French SS (2016). Physiological responses to salinity vary with proximity to the ocean in a coastal amphibian. Physiol Biochem Zool.

[38] Yue Y, Zhang M, Zhang J, Duan L, Li Z (2012). SOS1 gene overexpression increased salt tolerance in transgenic tobacco by maintaining a higher K^+^/Na^+^ ratio. J Plant Physiol.

[39] Wang N, Qi H, Qiao W, Shi J, Xu Q, Zhou H, Yan G, Huang Q (2017). Cotton (*Gossypium hirsutum* L.) genotypes with contrasting K^+^/Na^+^ ion homeostasis: implications for salinity tolerance. Acta Physiol Plant.

[40] Wang M, Zheng Q, Shen Q, Guo S (2013). The critical role of potassium in plant stress response. Int J Mol Sci.

[41] Wang N, Qi H, Su G, Yang J, Zhou H, Xu Q, Huang Q, Yan G (2017). Genotypic variations in ion homeostasis, photochemical efficiency and antioxidant capacity adjustment to salinity in cotton (*Gossypium hirsutum* L.). Soil Sci Plant Nutr.

[42] Meloni DA, Oliva MA, Martinez CA, Cambraia J (2003). Photosynthesis and activity of superoxide dismutase, peroxidase and glutathione reductase in cotton under salt stress. Environ Exp Bot.

[43] Jones JB (2016). *Hydroponics: a practical guide for the soilless grower*, CRC Press. Joseph, B., D. Jini and S. Sujatha. Development of salt stress-tolerant plants by gene manipulation of antioxidant enzymes. Asian J Agri Res.

[44] Oren A (2012). Salts and brines. *Ecology of cyanobacteria II.* Springer. Oueslati, S., N. Karray-Bouraoui, H. Attia, M. Rabhi, R. Ksouri and M. Lachaal. 2010. Physiological and antioxidant responses of *Mentha pulegium* (Pennyroyal) to salt stress. Acta Physiol Plant.

[45] Hameed A, Naseer S, Iqbal T, Syed H, Haq MA (2008). Effects of NaCl salinity on seedling growth, senescence, catalase and protease activities in two wheat genotypes differing in salt tolerance. Pak J Bot.

[46] Aslam M, Qureshi R, Ahmed N (1993). A rapid screening technique for salt tolerance in rice (*Oryza sativa* L.). Plant Soil.

[47] Ali Z, Khan AS, Khan IA, Azhar FM (2005). Heritability (h2b) estimates for NaCl tolerance in wheat (*Triticum aestivum* L.). J Agri Soc Sci.

[48] Ahmed BO, Inoue M, Moritani S (2010). Effect of saline water irrigation and manure application on the available water content, soil salinity, and growth of wheat. Agric Water Manag.

[49] Sheidai M (2008). Genetic and morphological variations induced by tissue culture in tetraploid cotton (*Gossypium hirsutum* L.). Acta Biologica Szegediensis.

[50] Flowers T, Yeo A (1995). Breeding for salinity resistance in crop plants: where next?. Func Plant Bio.

[51] Ahsan MZ, Majidano MS, Bhutto H, Soomro AW, Panhwar FH, Channa AR, Sial KB (2015). Genetic variability, coefficient of variance, heritability and genetic advance of some *Gossypium hirsutum* L. accessions. J Agric Sci.

[52] Flowers TJ, Galal HK, Bromham L (2010). Evolution of halophytes: multiple origins of salt tolerance in land plants. Func Plant Bio.

[53] Deinlein U, Stephan AB, Horie T, Luo W, Xu G, Schroeder JI (2014). Plant salt-tolerance mechanisms. Trends Plant Sci.

[54] Roff DA (2012). Evolutionary quantitative genetics, Springer Science & Business Media..

[55] Wu Z, Peng Y, Guo L, Li C (2010). Root colonization of encapsulated *Klebsiella oxytoca* Rs-5 on cotton plants and its promoting growth performance under salinity stress. Europ J Soil Biol.

[56] Dhamayanathi K, Manickam S, Rathinavel K (2010). Genetic variability studies in *Gossypium barbadense* L. genotypes for seed cotton yield and its yield components. Elec J Plant Breed.

[57] Zhang T, Qian N, Zhu X, Chen H, Wang S, Mei H, Zhang Y (2013). Variations and transmission of QTL alleles for yield and fiber qualities in upland cotton cultivars developed in China. PLoS ONE.

[58] Gama P, Inanaga S, Tanaka K, Nakazawa R (2007). Physiological response of common bean (*Phaseolus vulgaris* L.) seedlings to salinity stress. Afric J Biotech.

[59] Schachtman D, Munns R (1992). Sodium accumulation in leaves of *Triticum* species that differ in salt tolerance [wheat]. Aust J Plant Physiol.

[60] Schachtman D, Munns R (1992). Sodium accumulation in leaves of Triticum species that differ in salt tolerance. Func Plant Biol.

[61] Batool N, Shahzad A, Ilyas N (2014). Plants and salt stress. Int J Agri Crop Sci.

[62] Green S, Conatser M (2017). Assessment of soybean varieties in Arkansas for sensitivity to chloride injury. Soybean Research Studies.

[63] Lu W, Chu X, Li Y, Wang C, Guo X (2013). Cotton GhMKK1 induces the tolerance of salt and drought stress, and mediates defence responses to pathogen infection in transgenic *Nicotiana benthamiana*. PLoS ONE.

[64] Reni YP, Rao YK (2013). Genetic variability in soybean [*Glycine max* (L) Merrill]. Int. J. Plant. Anim Environ Sci.

[65] Ullah S, Khan AS, Raza A, Sadique S (2010). Gene action analysis of yield and yield related traits in spring wheat (*Triticum aestivum*). Int J Agric Biol.

[66] Sekhar L, Prakash B, Salimath P, Channayya P, Hiremath Sridevi O, Patil A (2010). Implications of heterosis and combining ability among productive single cross hybrids in tomato. Elect J Plant Breed.

[67] Sumathi P, Madineni S, Veerabadhiran P (2010). Genetic variability for different biometrical traits in pearl millet genotypes (*Pennisetum glaucum* LR BR.). Elect J Plant Breed.

[68] Sawarkar M, Solanke A, Mhasal G, Deshmukh S (2015). Combining ability and heterosis for seedcotton yield, its components and quality traits in *Gossypium hirsutum* L. Indian J Agric Res.

[69] Talpur MYM, Memon S, Memon S, Mari SN, Laghari S, Soomro ZA, Arain S, Dev W, A.A. Combining ability estimates from line x tester mating design in upland cotton. J Basic Appl Sci. 2016;12:378–82.

[70] Marulanda A, Azcón R, Chaumont F, Ruiz-Lozano JM, Aroca R (2010). Regulation of plasma membrane aquaporins by inoculation with a Bacillus megaterium strain in maize (*Zea mays*L.) plants under unstressed and salt-stressed conditions. Planta.

[71] Shiri M, Aliyev R, Choukan R (2010). Water stress effects on combining ability and gene action of yield and genetic properties of drought tolerance indices in maize. Res J Environ Sci.

[72] Ali Z, Salam A, Azhar FM, Khan IA (2007). Genotypic variation in salinity tolerance among spring and winter wheat (*Triticum aestivum* L.) accessions. S Afric J Bot.

[73] Salam A, Ali Z, Aslam M (2011). Sodium chloride tolerance in rice (*Oryza sativa* L.) at early seedling growth: genotypic variability, identification and selection. Pak J Bot.

[74] Macías JM, Caltzontzit MGL, Martínez ENR, W.a.N. Ortiz, A. Benavides Mendoza and P.M. Lagunes.  (2021). Enhancement tolerance in strawberry plants by iodine products application. Agron.

[75] Dong H, Kong X, Luo Z, Li W, Xin C (2010). Unequal salt distribution in the root zone increases growth and yield of cotton. Europ J Agron.

[76] Sampathkumar T, Pandian B, Mahimairaja S (2012). Soil moisture distribution and root charactersas influenced by deficit irrigation through drip system in cotton–maize cropping sequence. Agri Water Manag.

[77] Rahnama A, James RA, Poustini K, Munns R (2010). Stomatal conductance as a screen forosmotic stress tolerance in durum wheat growing in saline soil. Funct Plant Biol.

[78] Taïbi K, Taïbi F, Abderrahim LA, Ennajah A, Belkhodja M, Mulet JM (2016). Effect of saltstress on growth, chlorophyll content, lipid peroxidation and antioxidant defence systems in*Phaseolus vulgaris* L. S Afric J Bot.

[79] Sikder RK, Wang X, Jin D, Zhang H, Gui H, Dong Q, Pang N, Zhang X, Song M (2020). Screening and evaluation of reliable traits of upland cotton (Gossypium hirsutum L.) genotypes for salt tolerance at the seedling growth stage. J Cotton Res..

[80] Semida WM, Abd El-Mageed TA, Howladar SM, Rady MM (2016). Foliar-applied alphatocopherol enhances salt-tolerance in onion plants by improving antioxidant defence system. Aust J Crop Sci.

[81] Wang N, Qi H, Su G, Yang J, Zhou H, Xu Q, Huang Q, Yan G (2016). Genotypic variations inion homeostasis, photochemical efficiency and antioxidant capacity adjustment to salinity in cotton (*Gossypium hirsutum* L.). Soil Sci Plant Nutr.

[82] Sofo A, Scopa A, Nuzzaci M, Vitti A (2015). Ascorbate peroxidase and catalase activities and their genetic regulation in plants subjected to drought and salinity stresses. Int J Mol Sci.

[83] Nakano Y, Asada K (1981). Hydrogen peroxide is scavenged by ascorbate-specific peroxidase in spinach chloroplasts. Plant Cell Physiol.

[84] Dardalhon M, Kumar C, Iraqui I, Vernis L, Kienda G, Banach-Latapy A, He T, Chanet R, Faye G, Outten CE (2012). Redox-sensitive YFP sensors monitor dynamic nuclear and cytosolic glutathione redox changes. Free Radic Biol Med.

[85] Xiao W, Hao H, Xiaoqing L, Liang C, Chao L, Mingyu S, Fashui H (2008). Oxidative stress induced by lead in chloroplast of spinach. Biol Trace Elem Res.

[86] Mittova V, Volokita M, Guy M, Tal M (2000). Activities of SOD and the ascorbate-glutathione cycle enzymes in subcellular compartments in leaves and roots of the cultivated tomato and its wild salt-tolerant relative *Lycopersicon pennellii*. Physiol Plant.

[87] Ellouzi H, Hamed KB, Hernández I, Cela J, Müller M (2014). A comparative study of the early osmotic, ionic, redox and hormonal signaling response in leaves and roots of two halophytes and a glycophyte to salinity. Planta.

[88] Yang CW, Zhang ML, Liu J, Shi DC, Wang DL (2009). Effects of buffer capacity on growth, photosynthesis, and solute accumulation of a glycophyte (wheat) and a halophyte (*Chlorisvirgata*). Photosynthetica.

[89] El-Beltagi HS, Ahmad I, Basit A, Shehata WF, Hassan U, Shah ST, Haleema B, Jalal A, Amin R, Khalid MA, Noor F (2022). Ascorbic acid enhances growth and yield of sweet peppers (*Capsicumannum*) by mitigating salinity stress. Gesunde Pflanzen.

[90] Ksouri R, Ksouri WM, Jallali I, Debez A, Magné C (2012). Medicinal halophytes: potent source of health promoting biomolecules with medical, nutraceutical and food applications. Crit Rev Biotechnol.

[91] Sekmen AH, Ozgur R, Uzilday B, Turkan I (2014). Reactive oxygen species scavenging capacities of cotton (*Gossypium hirsutum* L.) cultivars under combined drought and heat induced oxidative stress. Environ Exp Bot.

[92] Frazier TP, Sun G, Burklew CE, Zhang B (2011). Salt and drought stresses induce the aberrant expression of microRNA genes in tobacco. Mol Biotech.

[93] Akhtar J, Saqib Z, Sarfraz M, Saleem I, Haq M (2010). Evaluating salt tolerant cotton genotypes at different levels of NaCl stress in solution and soil culture. Pak J Bot.

[94] Yao D, Zhang X, Zhao X, Liu C, Wang C, Zhang Z, Zhang C, Wei Q, Wang Q, Yan H (2011). Transcriptome analysis reveals salt-stress-regulated biological processes and key pathways in roots of cotton (*Gossypium hirsutum* L.). Genomics.

[95] Bublitz M, Poulsen H, Morth JP, Nissen P (2010). In and out of the cation pumps: P-type ATPase structure revisited. Curr Opinion Struc Bio.

[96] Bassil E, Tajima H, Liang YC, Ohto MA, Ushijima K, Nakano R, Esumi T, Coku A, Belmonte M, Blumwald E (2011). The *Arabidopsis* Na^+^/H^+^ antiporters NHX1 and NHX2 control vacuolar pH and K^+^ homeostasis to regulate growth, flower development, and reproduction. Plant Cell.

[97] Zhang L, Xi D, Li S, Gao Z, Zhao S, Shi J, Wu C, Guo X (2011). A cotton group C MAP kinase gene, GhMPK2, positively regulates salt and drought tolerance in tobacco. Plant Mol Biol.

[98] Shatil-Cohen A, Attia Z, Moshelion M (2011). Bundle-sheath cell regulation of xylem-mesophyll water transport via aquaporins under drought stress: a target of xylem-borne ABA?. Plant J.

[99] De Gara L, Locato V, Dipierro S, De Pinto MC (2010). Redox homeostasis in plants. The challenge of living with endogenous oxygen production. Resp Physio Neurobiol.

[100] Velikova V, Sharkey TD, Loreto F (2012). Stabilization of thylakoid membranes in isopreneemitting plants reduces formation of reactive oxygen species. Plant Signal Behav.

[101] Bokare AD, Choi W (2014). Review of iron-free Fenton-like systems for activating H2O2 in advanced oxidation processes. J Hazard Mat.

[102] Kim T-H, Böhmer M, Hu H, Nishimura N, Schroeder JI (2010). Guard cell signal transductionnetwork: advances in understanding abscisic acid, CO_2_, and Ca^2+^ signaling. Ann Rev Plant Bio.

[103] Hoagland DR, Arnon DI (1950). The water-culture method for growing plants without soil. Circular Calif Agric Exp Stn.

[104] Giri J (2011). Glycine betaine and abiotic stress tolerance in plants. Plant Signal Behav.

[105] Swaney DL, Beltrao P, Starita L, Guo A, Rush J, Fields S, Krogan NJ, Villén J (2013). Global analysis of phosphorylation and ubiquitylation cross-talk in protein degradation. Nat Methods.

[106] Sun SJ, Guo SQ, Yang X, Bao YM, Tang HJ, Sun H, Huang J, Zhang HS (2010). Functional analysis of a novel Cys2/His2-type zinc finger protein involved in salt tolerance in rice. J Exp Bot.

[107] Morran S, Eini O, Pyvovarenko T, Parent B, Singh R, Ismagul A, Eliby S, Shirley N, Langridgeand P, Lopato S (2011). Improvement of stress tolerance of wheat and barley by modulation of expression of DREB/CBF factors. Plant Biotech J.

[108] Gao S-Q, Chen M, Xia L-Q, Xiu H-J, Xu Z-S, Li L-C (2009). A cotton (*Gossypium hirsutum*) DRE-binding transcription factor gene, GhDREB, confers enhanced tolerance to drought, high salt, and freezing stresses in transgenic wheat. Plant Cell Rep.

[109] Jin LG, Li H, Liu JY (2010). Molecular characterization of three ethylene responsive element binding factor genes from cotton. J Integr Plant Biol.

[110] Wu C-A, Yang G-D, Meng Q-W, Zheng C-C (2004). The cotton GhNHX1 gene encoding a novel putative tonoplast Na+/H+ antiporter plays an important role in salt stress. Plant Cell Physiol.

[111] Xue T, Li X, Zhu W, Wu C, Yang G, Zheng C (2009). Cotton metallothionein GhMT3a, a reactive oxygen species scavenger, increased tolerance against abiotic stress in transgenic tobacco and yeast. J Exp Bot.

[112] Lu W, Chu X, Li Y, Wang C, Guo X (2013). Cotton GhMKK1 induces the tolerance of salt and drought stress, and mediates defence responses to pathogen infection in transgenic *Nicotiana benthamiana*. PLoS ONE.

[113] Meng C, Cai C, Zhang T, Guo W (2009). Characterization of six novel NAC genes and their responses to abiotic stresses in *Gossypium hirsutum* L. Plant Sci.

[114] Luo X, Wu J, Li Y, Nan Z, Guo X, Wang Y (2013). Synergistic effects of GhSOD1 and GhCAT1 overexpression in cotton chloroplasts on enhancing tolerance to methyl viologen and salt stresses. PLoS ONE.

[115] Li G, Tai F-J, Zheng Y, Luo J, Gong S-Y, Zhang Z-T (2010). Two cotton Cys2/His2-type zinc-finger proteins, GhDi19-1 and GhDi19-2, are involved in plant response to salt/drought stress and abscisic acid signaling. Plant Mol Biol.

[116] Zhou L, Wang N-N, Kong L, Gong S-Y, Li Y, Li X-B (2014). Molecular characterization of 26 cotton WRKY genes that are expressed differentially in tissues and are induced in seedlings under high salinity and osmotic stress. Plant Cell Tissue Organ Culture (PCTOC).

[117] Zhang F, Li S, Yang S, Wang L, Guo W (2015). Overexpression of a cotton annexin gene, GhAnn1, enhances drought and salt stress tolerance in transgenic cotton. Plant Mol Biol.

[118] Dhandapani G, Kanakachari M, Padmalatha KV, Phanindra MLV, Singh VK, Raghavendrarao S (2015). A gene encoding cold-circadian rhythm-RNA binding-like protein (CCR-like) from upland cotton (*Gossypium hirsutum* L.) confers tolerance to abiotic stresses in transgenic tobacco. Plant Mol. Biol. Rep..

[119] Wang W, Yuan Y, Yang C, Geng S, Sun Q, Long L (2016). Characterization, expression, and functional analysis of a novel NAC gene associated with resistance to verticillium wilt and abiotic stress in cotton. G3: Genes Genomes Genet..

[120] Liang C, Meng Z, Meng Z, Malik W, Yan R, Lwin KM (2016). GhABF2, a bZIP transcription factor, confers drought and salinity tolerance in cotton (*Gossypium hirsutum* L.). Sci. Rep..

[121] Rehman A, Wang N, Peng Z, He S, Zhao Z, Gao Q (2021). Identification of C2H2 subfamily ZAT genes in *Gossypium* species reveals GhZAT34 and GhZAT79 enhanced salt tolerance in *Arabidopsis* and cotton. Int J Biol Macromolecules.

[122] Serrano R, Rodriguez-Navarro A (2001). Ion homeostasis during salt stress in plants. Curr Opinion Cell Bio.

[123] Bose J, Rodrigo-Moreno A, Shabala S (2014). ROS homeostasis in halophytes in the context of salinity stress tolerance. J Exp Bot.

[124] Waisel Y (2012). Biology of halophytes. Elsevier.

[125] Radyukina N, Kartashov A, Ivanov YV, Shevyakova N, Kuznetsov VV (2007). Functioning of defense systems in halophytes and glycophytes under progressing salinity. Russian J Plant Physiol.

[126] Zhu YX, Gong HJ, Yin JL (2019). Role of silicon in mediating salt tolerance in plants: a review. Plants.

[127] Flowers T, Yeo A (1986). Ion relations of plants under drought and salinity. Func Plant Bio.

[128] Chaves MM, Flexas J, Pinheiro C (2009). Photosynthesis under drought and salt stress: regulation mechanisms from whole plant to cell. Ann Bot.

[129] Hernandez J, Jimenez A, Mullineaux P, Sevilia F (2000). Tolerance of pea (*Pisum sativum* L.) tolong-term salt stress is associated with induction of antioxidant defences. Plant Cell Environ.

[130] Hasegawa PM, Bressan RA, Zhu JK, Bohnert HJ (2000). Plant cellular and molecular responses to high salinity. Ann Rev Plant Bio.

[131] Parida AK, Das AB (2005). Salt tolerance and salinity effects on plants: a review. Ecotox Environ Safety.

[132] Adams P, Thomas JC, Vernon DM, Bohnert HJ, Jensen RG (1992). Distinct cellular and organismic responses to salt stress. Plant Cell Physiol.

[133] Tong Y-P, Kneer R, Zhu YG (2004). Vacuolar compartmentalization: a second-generation approach to engineering plants for phytoremediation. Trends Plant Sci.

[134] De Araújo SA, Silveira JA, Almeida TD, Rocha I, Morais DL, Viégas RA (2006). Salinity tolerance of halophyte *Atriplex nummularia* L. grown under increasing NaCl levels. Revista Brasileira de Engenharia Agrícola e Ambiental.

[135] Salama S, Trivedi S, Busheva M, Arafa A, Garab G, Erdei L (1994). Effects of NaCl salinity on growth, cation accumulation, chloroplast structure and function in wheat cultivars differing in salt tolerance. J Plant Physiol.

[136] Adams E, Shin R (2014). Transport, signaling, and homeostasis of potassium and sodium in plants. J Integrative Plant Bio.

[137] Sahu S, Das P, Ray M, Sabat SC (2010). Osmolyte modulated enhanced rice leaf catalase activity under salt-stress. Adv Biosci Biotech.

[138] Murphy MP, Holmgren A, Larsson N-G, Halliwell B, Chang CJ, Kalyanaraman B, Rhee SG, Thornalley PJ, Partridge L, Gems D (2011). Unraveling the biological roles of reactive oxygen species. Cell Metab.

[139] Ray PD, Huang B-W, Tsuji Y (2012). Reactive oxygen species (ROS) homeostasis and redox regulation in cellular signaling. Cellular signaling.

[140] Barrett G (2012). Chemistry and biochemistry of the amino acids, Springer Science & Business Media..

[141] Palmer BF. Regulation of potassium homeostasis. Clin J American Soc Neph CJN. 2014;10(6):1050–60. 10.2215/CJN.08580813 PMC4455213 24721891

[142] Johnson KL, Jones BJ, Bacic A, Schultz CJ (2003). The fasciclin-like arabinogalactan proteins of *Arabidopsis*. A multigene family of putative cell adhesion molecules. Plant Physiol.

[143] Wu CA, Yang GD, Meng QW, Zheng CC (2004). The Cotton GhNHX1 gene encoding a novel putative tonoplast Na^+^/H^+^ antiporter plays an important role in salt stress. Plant Cell Physiol.

[144] Xue T, Li X, Zhu W, Wu C, Yang G, Zheng C (2009). Cotton metallothionein GhMT3a, a reactive oxygen species scavenger, increased tolerance against abiotic stress in transgenic tobacco and yeast. J Exp Bot.

[145] Guo YH, Yu YP, Wang D, Wu CA, Yang GD, Huang JG, Zheng CC (2009). GhZFP1, a novel CCCH-type zinc finger protein from cotton, enhances salt stress tolerance and fungal disease resistance in transgenic tobacco by interacting with GZIRD21A and GZIPR5. New Phytol.

[146] Meng C, Cai C, Zhang T, Guo W (2009). Characterization of six novel NAC genes and their responses to abiotic stresses in *Gossypium hirsutum* L. Plant Sci.

[147] Gao SQ, Chen M, Xia LQ, Xiu HJ, Xu ZS, Li LC, Zhao CP, Cheng XG, Ma YZ (2009). A cotton (*Gossypium hirsutum*) DRE-binding transcription factor gene, GhDREB, confers enhanced tolerance to drought, high salt, and freezing stresses in transgenic wheat. Plant Cell Rep.

[148] Fan X, Guo Q, Xu P, Gong Y, Shu H, Yang Y, Ni W, Zhang X, Shen X (2015). Transcriptome-wide identification of salt-responsive members of the WRKY gene family in *Gossypium aridum*. PLoS ONE.

[149] Luo X, Wu J, Li Y, Nan Z, Guo X, Wang Y, Zhang A, Wang Z, Xia G, Tian Y (2013). Synergistic effects of GhSOD1 and GhCAT1 overexpression in cotton chloroplasts on enhancing tolerance to methyl viologen and salt stresses. PLoS ONE.

[150] Shelake RM, Kadam US, Kumar R, Pramanik D, Singh AK, Kim JY. Engineering drought and salinity tolerance traits in crops through CRISPR-mediated genome editing: Targets, tools, challenges, and perspectives. Plant Comm. 2022;14;3(6):100–17. 10.1016/j.xplc.2022.100417 PMC9700172 35927945

